# Supplementary material for: Complexome profiling on the Chlamydomonas lpa2 mutant reveals insights into PSII biogenesis and new PSII associated proteins
Source: J Exp Bot. 2021 Aug 26;73(1):245–62. doi: 10.1093/jxb/erab390 (PMC8730698; doi:10.1093/jxb/erab390)
Supplement: erab390_suppl_Supplementary_Dataset_S1 [file erab390_suppl_supplementary_dataset_s1.zip › Supplemental Dataset 1 - Excel List and all profiles/plots/METM_Cre06.g250200.html]

Trivial name: METM  
  
Euclidean distance: 26855.30  
Mean Intensity (WT): 4060.81  
Mean Intensity (Mut): 3479.04  
Distance: 6.61  
  
MapMan: metal handling.binding, chelation and storage;amino acid metabolism.synthesis.aspartate family.methionine.S-adenosylmethionine synthetase;amino acid metabolism.synthesis.aspartate family.methionine  
  
p value of intensity sums Welch test: 0.7953
